# Dehydration-mediated cluster formation of nanoparticles

**DOI:** 10.1038/srep11383

**Published:** 2015-06-16

**Authors:** Sungsook Ahn, Sang Joon Lee

**Affiliations:** 1 Biofluid and Biomimic Research Center; 2 Department of Mechanical Engineering, Pohang University of Science and Technology, Pohang, 790-784, Korea

## Abstract

Drying procedure is a powerful method to modulate the bottom-up assembly of basic building component. The initially weak attraction between the components screened in a solution strengthens as the solvent evaporates, organizing the components into structures. Drying is process-dependent, irreversible, and nonequilibrated, thus the mechanism and the dynamics are influenced by many factors. Therefore, the interaction of the solvent and the elements during the drying procedure as well as the resulting pattern formations are strongly related. Nonetheless still many things are open in questions in terms of their dynamics. In this study, nanoscale dehydration procedure is experimentally investigated using a nanoparticle (NP) model system. The role of water is verified in a single NP scale and the patterns of collective NP clusters are determined. Stepwise drying procedures are proposed based on the location from which water is removed. Effective water exodus from a unit NP surface enhances the attractive interaction in nanoscale and induces heterogeneous distribution in microscale. This study provides fundamental proof of systematic relation between the dehydration process and the resultant cluster patterns in hierarchical multiscales.

Water is one of the most important elements in nature and is indispensable for all biological systems to maintain life. As water is essential, so is dehydration. Cells and tissues able to maintain their life functions even in a dehydrated state for a long period by slowing down their metabolism. In addition to dried foods and household product applications, dehydrated human organs and blood potentially become available in the future for long-term preservation and easy transport^1^. The drying process is inherently far from equilibrium and exhibits complex transitory structures^2^,^3^ by evaporating solvent irreversibly^4^,^5^,^6^,^7^. As the solvent evaporates, the characteristic interactions occur among the components screened in a solution, initiating bottom-up assembly and further evolving into structural organization^4^. Although the dehydration-driven aggregation process can be explained with thermodynamic arguments^6^, it is in principle a nonequilibrium process^7^. A representative aggregation process caused by phase separation between dense nanocomponents and dilute solvent (or air) has been experimentally investigated^4^. However, the exact role of solvent fluctuations on the nanoscale drying procedure and conjunction with the resulting patterns have not been proved in detail^5^.

Nanoscale objects are critical components in the cellular lives^8^. In this point, bacteria^9^, macromolecules^10^, and nanoparticles (NPs)^11^ self-assemble in a similar manner, creating ordered structures with high precision. At this point, NP assembly plays an effective role as a model system for detailed observation of the molecular interactions. The concept established for NP association provides a roadmap for protein interactions, which are crystallized under slightly attractive interactions^12^. Crystallization is achieved by various attractive forces when interaction energy is adjusted within a certain range. In 2D assembly models, the micrometer-scale and submicrometer-scale spherical NPs suspended in liquids are readily formed into crystallized structures by sedimentation or drying processes. NP arrangements by the drying procedure are of significant use for broad applications, in this point the convective assembly is an easy and inexpensive method to create relatively large and periodically ordered structures. Through convective NP assembly occurring in the drying process, close-packed NP assemblies are usefully applied in various nano-coating technologies^13^,^14^. The NP assembly is of fundamental use to explain systematic dehydration procedures of a unit component that occurs in extensive ranges of living and artificial systems.

From a dynamic viewpoint, the evaporation process is a coarsening process where a spatially uniform NP domain is appropriately considered as a one-component fluid^4^. Then, union of the domains grows into a specific size (*r*) as a function of time (*t*) in the characteristic length scale based on the power law relation, *r* ~ *t*^α^. The coarsening process of NPs is self-similar and evolves through the dynamic process where the power law exponent (α) depends on the characteristic dynamical and physical constraints^15^. For example, when α ≈ 1/4, domain growth is solely mediated by cluster diffusion. Cluster mobility is inversely proportional to the size of the domain (*r*) and these dynamic behaviors dominate cluster growth at the intermediate state without solvent fluctuation or kinetic constraint^16^,^17^. Apart from the coalescence and shape fluctuations being visually similar, the scaling of domain size with time is also identical in most cases. Solvent evaporation induces the onset of the coarsening process, after which the governing mechanism and timescale of cluster growth are determined by the diffusion process of NPs (*D*). *D* is determined by the Stokes–Einstein relation mediated by the medium viscosity (*η*) and hydrodynamic size of a particle (*d*_*H*_): *D* = *k*_*B*_*T*/6*πηd*_*H*_. Although *d*_*H*_ is an important factor for dehydration, its effect on the dehydration process has not been clearly explained yet, especially from a dynamic viewpoint.

In this study, the role of the water in determining the clustering patterns of gold NPs (AuNPs) is experimentally investigated in terms of the drying kinetics. Dehydration of a unit AuNP is controlled by modulating the ligands on the AuNP with different water compatibility. The *d*_*H*_ values are dynamically changed according to the water−ligand relation. This population-modulated external compression and also stimuli-responsive internal shrinkage of ligand molecules enhance the attractive interaction of the AuNPs and induce a heterogeneous structural distribution in the entire system. These dynamic modulations of the AuNP surface are effective in controlling the dehydration procedure and the resultant pattern formation.

## Results

Bottom-up NP assembly model is designed as a basic building component of a continuum material. Using the NP model system, we investigate the 2D clustering mechanism in conjunction with time-dependent dehydration procedure. This determines the resultant pattern of the NP distribution in the designed system. We focus on hybrid nano-objects made of inorganic nanocores and organic ligands at the shell. This produces discretely integrated higher-order structures under nonequilibrium dynamic conditions by coordinating the growth, organization, and transformation of NP clusters. Citrate-covered gold nanoparticle (AuNP) solutions are prepared to develop the core of the NP at a fixed concentration. One of the main features of gold utilized in the present study is the ease of surface modification using chemisorption of thiol-containing species. Self-assembled thiolate monolayers on NPs have been previously verified in terms of effective ordering into characteristic pattern formation^18^,^19^,^20^,^21^. We utilize thiolate polyethylene oxide (PEO) and alkane with the designed composition, structure, and functional end groups incorporated into AuNP as ligands (Supporting Information).

**Figure 1:**
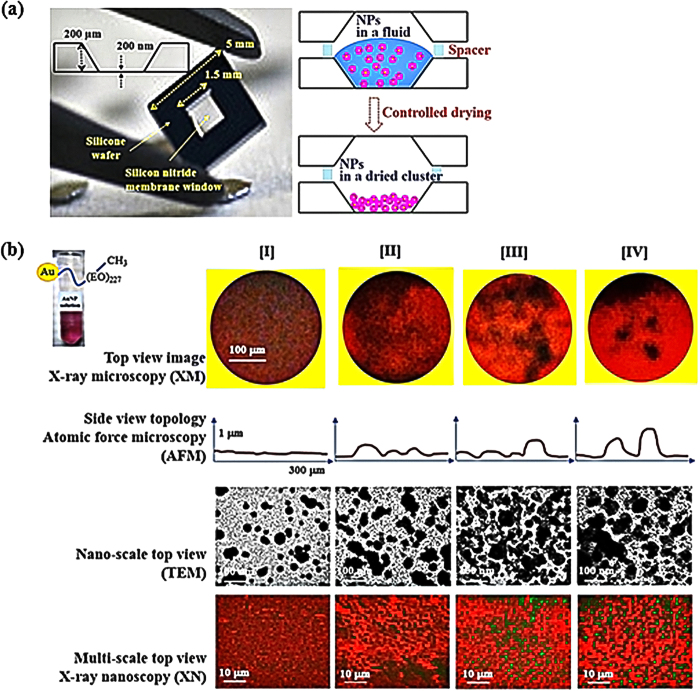
**(a)** Schematic of Si wafer sample holder containing the SiN_3_ membrane at the center. Ligand-conjugated AuNP solutions are loaded onto the membrane and dried under a given condition. For the standard condition, 35% humidity and 20 °C temperature are maintained. **(b)** Polyethylene glycol (PEG) 10000-linked AuNP images obtained by XM (top row), AFM (second row), TEM (third row), and XN (fourth row). Temperature is controlled at 20 °C [I], 40 °C [II], 60 °C [III], and 80 °C [IV] during the drying procedure. The diameter of the samples in the top view XM images is 300 μm. The depth of the samples for AFM detection is 10 μm. The reference bar indicates 100 nm for TEM images and 10 μm for XN images.

One considerable issue is the microscopic pattern formation driven by convection. When aqueous or organic droplets are dried, particles within the droplets are deposited in different patterns. Compositionally, the evaporation of aqueous droplets exhibits a coffee-ring effect, which accumulates particles at the rim, whereas most particles in nonaqueous droplets are deposited at the center. Geometrically, masking an evaporative droplet or film can diminish the microscopically modulated pattern formation^22^. In this study, a silicon (Si) wafer containing a silicon nitride (SiN_3_) membrane at the center is employed as a sample holder. Si wafer is used only for boundary and not for direct contact with the AuNP solution. The main reason to use SiN_3_ membrane for sample loading and covering is to hinder the coffee-ring effect^22^. The AuNP solution is loaded onto the SiN_3_ membrane. The contact angle of silicon nitride 28−30° indicates that this SiN_3_ membrane is hydrophilic^23^. From the obtained experimental results also the wetting of AuNP solution on the SiN_3_ membrane is high. Therefore, the water evaporation is not so fast and controlled by the designed way, minimizing the microscopic coffee-ring effect. The microscopic convection-driven pattern formation is reduced, then relatively homogeneous NP-driven clusters are favorably formed. Another considerable issue is the concentration of NPs on the substrate. The increase in coverage (NP concentration) induces an increase in the characteristic domain sizes under the condition of liquid–vapor coexistence. Aqueous AuNP solution placed on SiN_3_ membrane of Si wafer holder is dehydrated under controlled experimental conditions (Fig. 1(a), left). The other Si wafer holder is used as a cover. Between these two wafers, a teflon O-ring of 100 μm thickness is inserted with intermittent scissions as a spacer. This controls the aeration for controlled dehydration not for sealing (Fig. 1(a), right). This geometry is exactly the same for all of the employed samples. All the experiments have been performed in the designed chamber under constant temperature and humidity condition (25 °C and 35% humidity). We do not actually change the experimental conditions, but constant condition is carefully maintained. The characteristic time for water evaporation (*τ*) and NP diffusion (*τ*_*D*_) increases along with the increase in coverage. The domain size (*R*) is determined by the NP mobility (*D*) and elapsed time until the growth stops (*τ*_*s*_). At a low coverage condition of small amount of NPs, the aggregation process takes a long time (large *τ*_*s*_/*τ*). At an appropriately high coverage condition, larger aggregates appear along with the increase in *τ*_*s*_/*τ*.

In this study, colloidal AuNPs of 20 nm in average diameter are dispersed in aqueous solution for which the standard concentration of the AuNP is controlled at approximately 2.4 × 10^12^ AuNPs/mL. Homogeneity is aimed in the patterns formed by AuNP distributions on the SiN_3_ membrane under a designed condition as a result of the drying procedure. As a representative pattern formation, the selected AuNP aqueous solutions are dried under the designed conditions as depicted in Fig. 1(b): AuNPs coated with methyl end-capped PEO (AuNP–(EO)_*n*_–CH_3_, *n* = 227). Depending on the drying condition (20 °C [I], 40 °C [II], 60 °C [III] and 80 °C [IV]), the spatial distribution of the AuNPs is changed. The increase in temperature induces phase separation of PEO from water, which dominates AuNP aggregation and enhances the heterogeneity of the entire system (discussed in detail at a later part). Top view images are obtained by X-ray microscopy (XM) where the AuNP clusters are expressed as black parts. By contrast, side view topological profiles are obtained by atomic force microscopy (AFM), where the extruded parts indicate the AuNP clusters. Therefore, the geometrical and topological factors are matched as confirmed by the top view and side view images. The transmission electron microscopy (TEM) and X-ray nanoscopy (XN) images also verify the results. Therefore, the results of AFM are directly related with the XM, XN and TEM results. The value of the AFM results indicates longitudinal bumpy state of the clusters of 1 μm-thick height in the images. So as we believe that the black clusters are higher than other white image both in X-ray imaging and TEM. The side view topological profiles quantitatively exhibit the degree of distortions in the assembled materials induced by heterogeneous distribution of AuNPs. Heterogeneity increases from state [I] to [IV]. The homogeneous distribution of AuNPs [I] indicates that the energy is balanced and the shape of the material is maintained. By contrast, the heterogeneously aggregated AuNPs [IV] represent prominently distorted spatial distribution.

**Figure 2:**
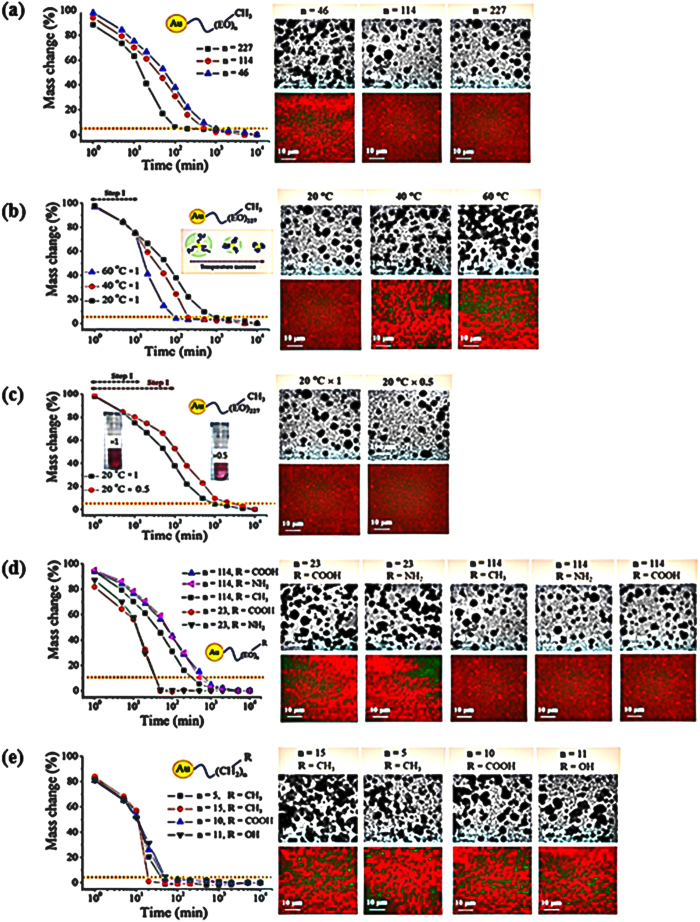
Variations of mass change (left graph) and cluster images (right graph) as shown in the TEM (upper images) and XN images (lower images) **(a)** Three methyl end-capped PEO systems with *n* = 46, *n* = 114, and *n* = 227 are compared. Shorter PEO of *n* = 46 exhibits faster drying kinetics (left graph), more aggregation, and increased heterogeneity compared with *n* = 114 and *n* = 227 systems. **(b)** Increase in temperature from 20 °C to 40 °C to 60 °C at a fixed concentration ( × 1) of *n* = 227 systems. **(c)** Two different AuNP concentrations of × 0.5 and × 1 at a fixed temperature of 20 °C. **(d)** The PEO-based AuNPs with the designed functional end groups. The number of the EO unit is controlled to be *n* = 23 and *n* = 114 with base (NH_2_), acid (COOH), and hydrophobic methyl (CH_3_) end groups. Under controlled drying conditions, the shorter EO unit of the fast drying period generates more aggregates and forms AuNP clusters, as shown in the TEM and XN images. **(e)** Alkyl-based AuNPs with the designed functional end groups. The length of the alkyl chains is controlled to be *n* = 5, *n* = 10, *n* = 11, and *n* = 15 with acid (COOH), hydrophilic hydroxyl (OH), and hydrophobic methyl (CH_3_) end groups. Under controlled drying conditions, the longer alkyl chain of the fast drying period generates more aggregates and forms AuNP clusters.

The amount of water bound to macromolecules in a solution is estimated by normalized mass change (%): *M* = [(*M*_0_ − *M*_*t*_)/(*M*_0_ − *M*_*f*_)] × 100, where *M*_0_ is the initial mass, *M*_*f*_ is the final mass, and *M*_*t*_ is the mass at time *t*. The average mass of one sample is close to 200 mg with 100 mg of AuNP sample loading. Each point in the graph has been obtained by averaging the mass of 20 samples under the same condition. The drying kinetics is plotted by semilogarithmic curves to estimate α in the following power law relation: *M* ~ *t*^−α^. The final mass (*M*_*f*_) is determined at the equilibrium time (*t*_*eq*_) from which the mass does not change anymore under the given condition. This does not mean a complete sample dry but equilibrated state (We do not discuss complete water elimination from the PEO molecules in this study). As displayed in Fig. 2, *M*_*f*_ is determined from the equilibrium mass without any further mass change, expressed by a dotted line in each graph in consideration of 5% error. After seven days of total drying procedure performed under 35% humidity and 20 °C temperature, TEM and XN images are obtained (Supporting Information). The variation of drying profiles is investigated for a series of samples with different hydrophilicity and end groups under the designed drying conditions. The TEM (top) and XN (bottom) images verify the final results of the AuNP clusters in nano-scale. Acceleration of the drying process prominently induces aggregated AuNPs to form clusters.

For the systematic investigation of the drying process mediated by ligand molecules, temporal variations of mass are determined in the methyl end-capped PEO series with three different molecular weights of *n* = 46, *n* = 114, and *n* = 227 at 20 °C of standard concentration (×1) as displayed in Fig. 2(a). Depending on the length of the PEOs anchored on AuNPs, the drying patterns are distinctively differentiated. The shorter EO unit exhibits a faster drying than the longer EO units. This reflects that the longer EO units can hold water more effectively. As shown in the TEM and XN images at the right side, the fast drying system of *n* = 46 exhibits more aggregation than other systems of *n* = 114 and *n* = 227. The nanoscale TEM and zoomed-out XN images of *n* = 114 and *n* = 227 exhibit a relatively homogeneous dispersion of AuNPs. The mass change rate is significantly diversified depending on the PEO type.

To identify the role of each drying procedure, the dynamics of drying is modified and its relation with the resultant cluster pattern is experimentally investigated. First, temperature variation is investigated for a PEO of *n* = 227 in Fig. 2(b). The solubility of PEO is decreased when the temperature is greater than the lower critical solution temperature (LCST)^24^,^25^. Considering this fact, we designed the drying process for AuNP of *n* = 227 using three different temperatures of 20, 40, and 60 °C. The first peak *q*_*I*_ of this AuNP of *n* = 227 shifts toward the higher *q* region with the temperature increase, which indicates the temperature-induced shrinkage of the PEO shell (Fig. S4). Considering that bulk water evaporation is dominant at step I, heating is applied in step II with constant humidity of 35%, by which the PEO shrinkage effect is exclusively observed. As the temperature increases from 20 °C to 60 °C, the drying rate accelerates. The resultant cluster patterns are compared at the right side. The temperature increase produces more aggregated clusters thus more heterogeneously distributed AuNPs as observed in the TEM and XN images. In addition to the evaporation of water molecules at the standard condition, physically promoted shrinkage of the PEO shell induced by stimuli responsiveness further accelerates the drying process and enhances the AuNP aggregation. Therefore, the ligand–water interaction dominates the drying kinetics and resultant aggregation of AuNPs. In addition, the drying procedure is independent of the driving source, either by density-controlled congestion (external compression) or by responsive contraction of ligand molecules (internal shrinkage).

Next, initial step of drying is modified by diluting the standard AuNP solution such that the number of AuNPs in the solution is half (×0.5) that of the standard (×1) in Fig. 2(c). The evaporation of water molecules in bulk and breakage of water-to-water interaction takes a longer time. The elapsed time for initial drying step is extended for the diluted ×0.5 solution than that of standard because of the presence of a large amount of water. A smaller amount of NPs in the solution takes a longer time to be clustered (large *τ*_*s*_/*τ)*. The AuNP solutions of the designed initial concentration undergo several steps to be further concentrated by the dehydration procedure. The drying rate is prominently delayed compared with that of the standard (×1) solution. Even though delayed, the slope of the ×0.5 solution is similar to that of the ×1 solution (i.e., α is similar). This drying profile modification just by changing the *τ*_*s*_/*τ* does not influence the resulting clustering of AuNPs.

The effects of unit number (*n*) and end group^26^ of ethylene oxide (EO) are graphically summarized in Fig. 2(d). The EO unit number is changed from *n* = 23 to *n* = 114. The end group effects are compared for each EO unit. A short EO unit of *n* = 23 accelerates the drying process significantly. This finding implies that the water-holding ability of a longer EO unit delays mass change. Generally, the water trapped by EO has two types of states: free water molecules in bulk and water molecules bounded to the EO units. The ability of escaping of water from the solution contributes to the mass evolution during the drying process, which depends on different EO unit number. In the same concept but with a different mechanism, the incorporation of hydrophilic cationic (–NH_2_) and anionic (–COOH) end groups into ligands noticeably delays mass change compared with the case of the hydrophobic methyl (–CH_3_) end group incorporation in Fig. 2(d). The results with alkyl chains of designed length and end groups anchored on an AuNP are investigated in Fig. 2(e). Compared with the EO-based ligands, all of the alkyl-based ligands exhibit faster drying profiles. The hydrophobic methyl end-capped pentadecane results in the fastest drying process, whereas the ligands of short pentane or hydrophilic end groups result in a slightly slower drying process. Therefore, water compatibility of the ligands has a critical influence on the drying kinetics. In addition, the resultant clusters show that the fast drying process enhances the aggregation of AuNPs, thus increasing the heterogeneity of the entire system.

**Figure 3:**
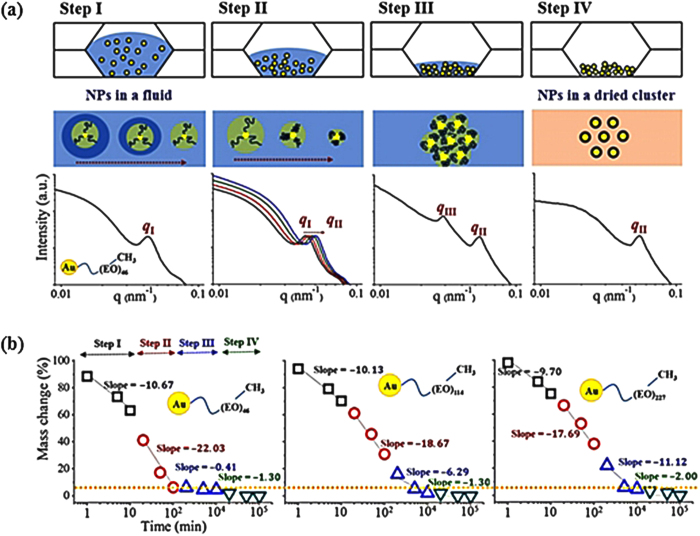
**(a)** Four steps of the drying procedure are proposed and called steps I to IV. The first line depicts the concept of the four drying steps of AuNP aqueous solutions positioned between SiN_3_ membranes. The other lines depict the proposed models. Each drying step is confirmed by the representative SAXS profile for a methyl end-capped *n* = 46 PEO-conjugated AuNP system. At step I, the hydrodynamic size (*D*_*H*_) of a single AuNP (*q*_*I*_) in dilute solution is almost similar. At step II, the *q*_*I*_ shifts to a higher *q* region until it reaches the minimum (*q*_*II*_). This finding indicates shrinkage of the shell part of AuNP. At step III, a structure is formed in the highly concentrated viscous solutions induced by the aggregated AuNP clusters expressed by a peak (*q*_*III*_). At step IV, completely dried AuNP powders exhibit no further aggregated structures. **(b)** Drying kinetics of the three PEO systems in (a) are analyzed based on steps I to IV categorized based on the SAXS profiles. The most prominent difference occurs at step II.

Based on the aforementioned mass change results, four drying steps are proposed according to the drying time in Fig. 3(a). Each step is verified by small-angle X-ray scattering (SAXS). The average distance between the particulate objects is evaluated by the distance relation, 2π/q*, where q* is the peak position of the scattering vector *q*. The top line depicts the drying process of AuNPs between SiN_3_ membranes with continuous decrease in the mass by water evaporation. The middle line shows an assembly model proposed for the AuNPs and clusters. Representative SAXS results at each step are shown in the bottom line with the representative AuNPs covered with methyl end-capped PEO (*n* = 46). At a dilute condition of step I (the structure factor approaches unity and only the form factor is valid), the hydrodynamic size (*d*_*H*_) of a single AuNP is exclusively determined by the position of the peak *q*_*I*_. Although the mass of the entire AuNP solution continuously decreases during the drying procedure, the position of the peak *q*_*I*_ is nearly similar up to the next step without generating any detectable structure factor. The results show that only water molecules located away from the AuNP surface are diminished by the step I drying procedure. By contrast, those captured by the ligands on the AuNP surface are not changed noticeably. This finding indicates that water-to-air interaction in bulk is dominant, compared with water-to-ligand interaction on the AuNP surface. Therefore, during step I, water molecules are evaporated mainly by disconnecting the water-to-water interaction in the bulk. Thus, *d*_*H*_ value of the AuNP is almost fixed. In addition, few interactions between the fully hydrated AuNPs are observed, which lead to an almost unchanged peak *q*_*I*_, as depicted in the proposed model at the middle line of Fig. 3. At step II, the drying procedure causes conformational change of ligand molecules on the AuNP. As a result, the peak shifts toward the higher *q* region, starting from the initial *q*_*I*_ to the maximum *q*_*II*_. Thereafter, individual AuNPs in the shrunken state are aggregated during step III, forming structural assemblies detected by a peak *q*_*III*_ at a lower *q* region than the position of *q*_*II*_. Further drying of the system at step IV generates breakage (or modification or maintenance, depending on the case) of the aggregated *q*_*III*_ structure, while the smallest size of the completely dried single AuNP is maintained at *q*_*II*_ in most cases. The absence of water in step IV implies that the movement of AuNPs does not have any physical meaning.

Based on the proposed four drying steps, each drying time domain is categorized by mass change for three PEO systems in Fig. 3(b). The obtained *q* values by SAXS for each drying step are included in the Supporting Information (Fig. S3). The *q*_*I*_ and *q*_*II*_ values decrease when the molecular weight increases from *n* = 46 to *n* = 227. This finding is attributed to the larger *d*_*H*_ of the longer PEO-anchored AuNP and the larger structural assembly induced by the drying process. Although the absolute value and the mass change rate (the slope implies α) for each system are different, the times that elapsed to reach each drying step are almost similar for the three PEO systems. The slope α in the semilogarithmic plot is nearly similar at step I for the three PEO systems. By contrast, the α at step II is higher for *n* = 46 than those for *n* = 114 and *n* = 227. However, at step III, the slope α is higher thus, water evaporation in the clustered system is more accelerated for the longer PEO. Therefore, before reaching step IV, the shorter and longer PEOs are fully dehydrated by alternative effectiveness in drying. The α at step IV is approximately zero and lacks physical meaning in terms of AuNP arrangement. Nonetheless, there is a clear mass decreases for all the systems.

**Figure 4:**
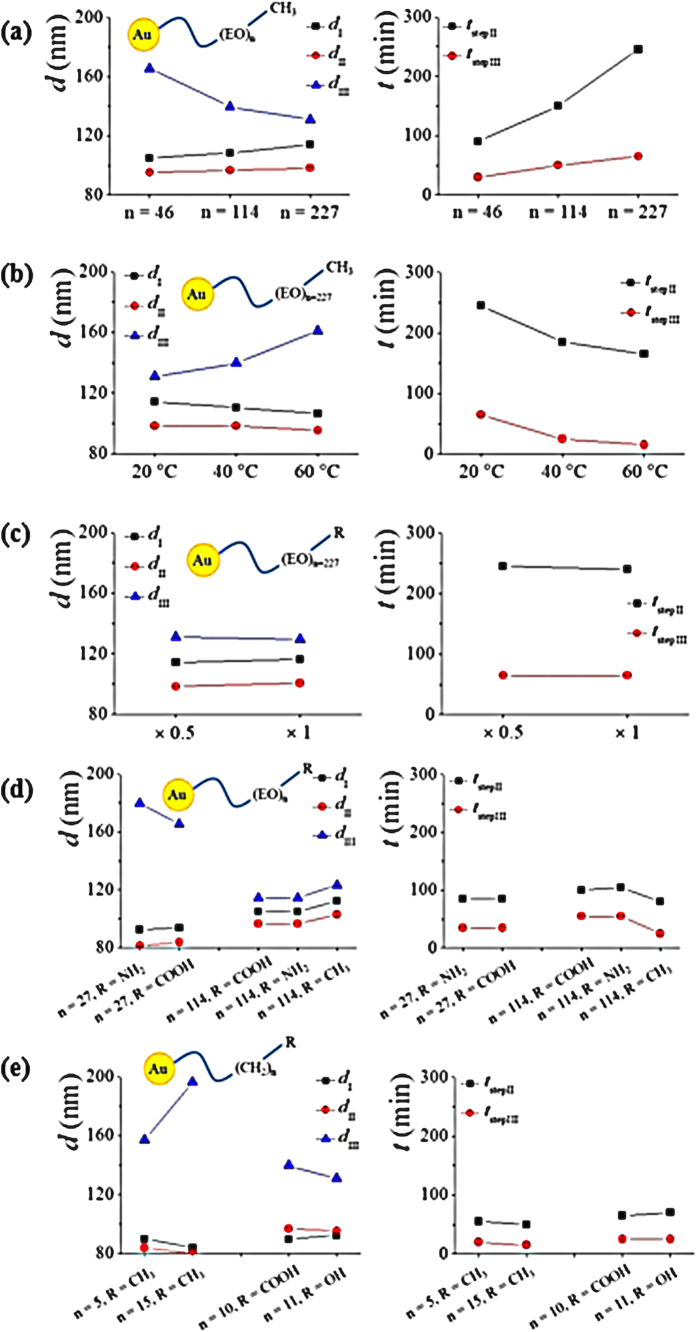
Cluster size variation and time relation. dI, dII and dIII are determined by the SAXS peaks of qI, qII and qIII respectively. **(a)** Three methyl end-capped PEO systems with *n* = 46, *n* = 114, and *n* = 227 are compared. **(b)** Increase in temperature from 20 °C to 40 °C to 60 °C at a fixed concentration ( × 1) of *n* = 227 systems. **(c)** Two different AuNP concentrations of × 0.5 and × 1 at a fixed temperature of 20 °C. **(d)** The number of the EO unit is controlled to be *n* = 23 and *n* = 114 with base (NH_2_), acid (COOH). **(e)** The length of the alkyl chains is controlled to be *n* = 5, *n* = 10, *n* = 11, and *n* = 15 with acid (COOH), hydrophilic hydroxyl (OH), and hydrophobic methyl (CH_3_) end groups.

The mass change are comparatively investigated with the SAXS results. The AuNP size and further formed cluster sizes expressed by *d*_I_, *d*_II_, and *d*_III_, which are obtained by the characteristic peak of *q*_I_, *q*_II_ and *q*_III_ in SAXS profiles (left side graphs). The elapsed time for step II and step III are expressed as *t*_stepII_ and *t*_stepIII_ (right side graphs). The results are consistent with the mass change results, therefore the suggested mechanism in Fig. 3(a) is verified. For all the systems, there is a shrinkage of the AuNP during the step II. This is verified by the smaller *d*_II_ than *d*_I_. Further cluster formations are confirmed by the significantly increased *d*_III_. The elapsed time of step II are all the time longer than the step III, which indicates that the shrinkage procedure takes longer time than cluster formation. in addition, slow procedure prominently leads to smaller cluster while fast procedure generates larger clusters. In terms of the EO length effect in Fig. 4(a), short EO unit generates large cluster for the short time period. In the same concept increased temperature in Fig. 4(b), generates fast clustering during the short time. AuNP solution concentration effect does not generate any mechanistic difference confirmed by the results as displayed in Fig. 4(c). EO unit and hydrophobic end-group contribute to the prominent increase in *d* which are matched with the decrease in the elapsed time *t*, for EO-based AuNPs in Fig. 4(d). On the same concept, longer alkyl chain length increases the cluster size significantly with relatively short time in Fig. 4(e). Similar length of alkyl chain length and hydrophilic end-groups shows similar results, this means that those factors are decisive factor for drying mechanism.

**Figure 5:**
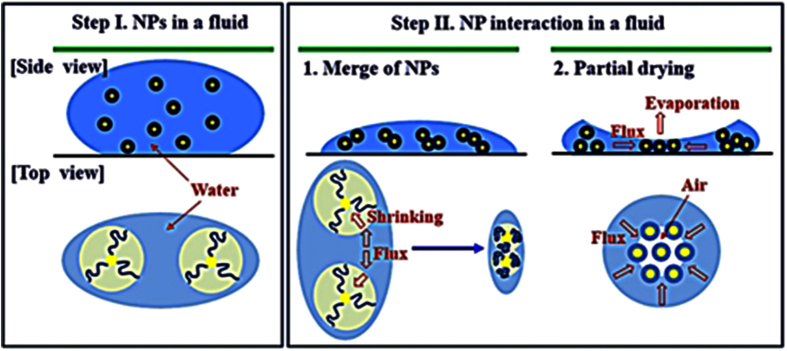
NP cluster model. At step I, fully hydrated NPs are dispersed in water. At step II, two different mechanisms are proposed. First, NPs are merged and congested based on the dehydration procedure. With shrinkage of the ligands, a fluid flux is formed. Second, the partially dehydrated NPs are exposed to air to which fluxes are formed. These two interactive fluxes lead to the formation of close-packed NPs and clusters.

The final stage of the drying process where there is an air-water meniscus formation between the solid particles^27^. Here, the surface molecule-induced shrinking process in an individual AuNP is important in the drying process that occurs mainly at step II. At this point, the less hydrophilic shorter PEO *n* = 46 is advantageous for more effective water evaporation from the ligand molecule than the longer PEO, which leads to increased clustering and heterogeneous distribution of AuNPs. As illustrated in Fig. 5, at step II, when NPs are shrunk (merge of NPs) or exposed to air (partial drying) by dehydration, a converging flux to these shrunk/exposed NP domains exists^27^, which promotes the AuNP clusters. Therefore, rather than step I in which water molecules are evaporated in bulk, step II is more important in arranging the NP components, thereby dominating the final patterns of the clusters. Thereafter, the particle-to-particle distance becomes closer by effective water evaporation by shrinkage of an individual AuNP or exposure of AuNPs to air. The pattern of clusters is determined at step III with the effective formation of clustered structures dominating the heterogeneity of the entire system.

## Discussion

NP aggregation by dehydration follows irreversible coagulation processes to form clusters at the final stage^28^. The aggregation depends on the drying process especially on the elapsed time to take NPs into a contact. The fractal-like appearance of the cluster structures originates in dynamics that are locally similar to diffusion-limited aggregation hereby particles undergoing a random walk by Brownian motion^29^. Attachment of NPs to the growing domains is nearly irreversible because the front of a vapor nucleus passed by. Strictly irreversible binding of random walkers has been observed in several experiments to generate fractal structures^30^,^31^. For an irreversible association, the colloidal solid particles are kinetically trapped in their initial configuration. This configuration is typically expressed by the fractal dimension ( *f* ) based on the relation between mass (*M*) and size (*R*): *M* ~ *R*^*f*^. For fast-dried clusters, a highly compacted nanoscale aggregation represents a large *f*, while a highly heterogeneous distribution of microscale aggregation reflects a small *f*. By contrast, an opposite description is employed to express slowly dried clusters. Therefore, in description of NP cluster formation *f* value is a function of the scale factor and also a clustering time (τ_s_). These aggregation phenomena are supported by multiscale imaging results in which highly aggregated AuNPs in nanoscale (observed in the TEM images) lead to ramified porous structures in microscale (shown in the XN images).

In microscopic viewpoint, the driving force for NP assembly instantaneously varies based on their interparticle positions at a given instant. If NPs are sufficiently mobile to track vapor nuclei growth by exposure to air, then the pattern of the NP aggregate is determined by the structural history of evaporation. In particular, after a long period, the NP domains are roughly located at the intersection lines of colliding vapor nuclei, leading to network-like morphologies. This assembly mechanism resembles in many points the Marangoni effect^30^. A network is formed when the edges of the NP domain are effectively frozen after evaporation. This implies that the NP aggregation essentially halts when vapor nuclei collide. Each network structure represents an independent nucleation event in which its front pushes NPs to the domain boundaries. The shapes of terminal structures in these trajectories are determined primarily by the relative timescales of the evaporation process and NP motion, *τ*_*D*_ = *ζ*^2^/*D*, where *D* is the NP diffusion coefficient in the solution and *ζ* is the correlation length of the solvent. For sufficiently large *τ*/*τ*_*D*_, the edges of the NP domains are rearranged before the evaporation of the solvent renders them immobile. The smooth edges of rod-like aggregates and network boundaries are attributed to such relaxation. By contrast, when *τ*/*τ*_*D*_ is small, the domain edges are effectively frozen as they are formed, which results in highly ramified network structures.

Compared with the unstructured particle colony, the ordering of monodispersed particles in a liquid permits large-scale local free space available for each particle, which causes high-level translational entropy. As the volume fraction of spherical particles approaches 55% the ordering procedure leads to a phase transition by the enhanced total entropy of the system. On the other hand, the spherical particles are crystallized when their free volume is restricted below a certain threshold. These entropy-driven crystallization of monodispersed spherical particles has been investigated in detail theoretically^32^ and experimentally^33^,^34^. However, this phenomenon occurs when the interactions between spherical particles are repulsive, inducing their rearrangement^35^. During the dehydration process, the designed AuNPs are continuously concentrated thus they are externally compressed. The particles are inherently suggested to be attractive by induced flux formation. These processes are thus different from the aforementioned typical entropy-driven crystallization. The ligand-modulated drying process induces systematic clustering by water-mediated molecular movements and importantly determines the resultant cluster patterns in a dynamic way. Nonetheless, similar to the entropy-driven crystallization, the shrinkage of the ligands significantly modulate the volume fraction of the particle system. And this critically also influences the entropy and further resulting patterns.

The temporal evolution of NP aggregation can be quantitatively compared. The use of different solvents and various evaporation conditions adjust the chemical potential (*μ*), NP mobility (*D*), and attractive energy between the components, especially the NP-to-liquid strength (*ε*_*nl*_) and the liquid-to-liquid strength (*ε*_*ll*_). The cluster mobility (*D*_cluster_ not *D* for a single NP) is inversely proportional to the cluster size (*r*), thus the cluster diffusion is expressed by the time-independent size distribution. When the evaporation process has a relatively slow NP mobility, the mean squared displacement (*L*) of an identified NP for a designed physical timescale (∆*t*) follows the relation: *L* ~ 4*D∆t* ~ 1/*r*. These conditional factors has been mostly suggested for the fixed-sized hard spheres. The designed AuNP dehydration in this study is however, modulated by changing the *d*_*H*_, therefore, additional dynamic modulation is expected. Nonetheless, from the fact that delayed dehydration (large *∆t*) induces small cluster size (*r*), the aforementioned general relation seems to be similarly applied to the designed system. Therefore, the suggested general relations are extended to the cases of variable particle size.

In terms of the ligand performance linked on the AuNP surface, the thermodynamics of ligand suspending in solutions needs to be considered in detail. The phase separation of molecular (ligand) solution covering the AuNP surface generates the attractive interaction among the AuNPs, in addition to the coupling between two phase transitions in solvent and NP of different density. The self-assembled morphology of NP clusters is largely affected by heterogeneous or homogeneous evaporation and NP domain wetting. Without solvent fluctuation, the NP assembly is related to coarsening that has been studied in terms of spinodal decomposition^3^,^15^. When evaporation is spatially homogeneous, simple coarsening occurs because solvent deficiency drives the aggregation of NPs. As long as the boundaries are wet by the solvent, the NP boundaries remain fluxional throughout the growth procedure. A wetting layer remains in a thermally stable state in the limit of homogeneous drying, which satisfies the condition: *μ* > *k*_*B*_*T* − *ε*_*nl*_ − *ε*_*ll*_, where *k*_*B*_*T* corresponds to the energetic cost of moving a NP into the surrounding solvent, *k*_*B*_ is the Boltzmann constant and *T* is the temperature. The growing domains are rearranged and conglomerated when the solvent is near the spinodal metastable state, *μ* ≈ *μ*_*sp*_(*T*). Between the binodal and spinodal lines, −2*ε*_*ll*_ > *μ* > *μ*_*sp*_(*T*), the solvent remains locally metastable on the NP surface. This is the condition where the phase change is driven by nucleation and aeration. By contrast, when evaporation is strongly heterogeneous in space, the dynamic self-assembly behaviors are dramatically differentiated. Depending on the ligand molecule type anchored on an AuNP, the phase separation step characteristically progresses by the dynamic water evaporation. At isothermal condition of this study, the phase separation is mainly driven by the concentrating procedure of the molecules. Dynamic water evaporation changes the chemical potential (*μ*), NP mobility (*D*), and attractive energy between the components, especially the NP-to-liquid strength (*ε*_*nl*_) and the liquid-to-liquid strength (*ε*_*ll*_), the cluster mobility (*D*_cluster_) of a given size (*r*). Therefore, stability is changed along with the drying procedure. According to the time for the water loss displayed in Fig. 4, short EO unit, long alkyl chain and hydrophobic end-group promote the size decrease of an individual AuNP by water loss of ligands followed by the cluster size increase. For those fast drying systems seem to take spinodal phase separation more effectively. On the other hand, the slow drying systems goes to the between the bimodal and spinodal, so that there is water on the AuNP surface for a long time.

With its current practical application and further potential significance, dehydration may become a more important issue than expected in extensive scientific and industrial fields. In previous computational studies, the folding and association of proteins have been investigated in terms of hydrophobic interfacial interactions^36^,^37^,^38^, focusing on the use of hydrophobic dewetting as an essential driving force in biological phenomena^38^,^39^,^40^,^41^. From another viewpoint, hydrophilic interfaces used for assembly are of significant interest by considering the fact that an average 70% of the interfacial residues of protein complexes are hydrophilic^42^,^43^,^44^. Although hydrophilic protein interfaces are often involved in the formation of protein complexes, the assembly mechanism of such surfaces has been studied only a few. Either hydrophobic or hydrophilic, the molecular interaction with water has been the focusing point of discussion in terms of biomolecular interaction and their functioning issues. We suggest that the relation between the nanoscale components and solvent is crucial in terms of dynamic flux generation.

In summary, the dehydration procedure investigated by the proposed NP model in this study provides fundamental insights into the dynamic aspects of the drying procedures and the role of water interaction with nanoscale components. It can be extended into the structural heterogeneity issues of biological systems, such as protein crystallization. In terms of the partial drying kinetic mechanism by which a fluid flux within a droplet is formed, we propose the nanoscale dynamic merging of NPs by inducing shrinkage of the ligands on the NP surface. This is confirmed by the mass change and size variation by SAXS results. The result is suggested to be induced by the contribution of the nanoscale flux formation. Rather than the hydrophobic or hydrophilic forces, which have been reported to be dominant for such dynamic phenomena, the detachment procedure of water molecules from unit components is proposed to be critical in nanoscale dehydration.

## Additional Information

**How to cite this article**: Ahn, S. and Joon Lee, S. Dehydration-mediated cluster formation of nanoparticles. *Sci. Rep.* **5**, 11383; doi: 10.1038/srep11383 (2015).

## Supplementary Material

Supplementary Information
