# Supplementary material for: Dehydration-mediated cluster formation of nanoparticles
Source: Sci Rep. 2015 Jun 16;5:11383. doi: 10.1038/srep11383 (PMC4468418; doi:10.1038/srep11383)
Supplement: Supplementary Information [file srep11383-s1.doc]

**Dehydration-mediated cluster formation of nanoparticles**

Sungsook Ahn^1,2^ and Sang Joon Lee^1,2^,*

^1^Biofluid and Biomimic Research Center, ^2^Department of Mechanical Engineering,

Pohang University of Science and Technology, Pohang, 790-784, Korea.

*Correspondence

Tel: +82-54-279-2169, Fax: +82-54-279- 3199, E-mail: sjlee@postech.ac.kr

**Supporting information**

**Preparation of spherical AuNPs of 20 nm in average diameter.** To prepare AuNPs, gold chloride (III) trihydrate (HAuCl_4_·3H_2_O) is dissolved in deionized (DI) Milli-Q water (1.0 × 10^−3^ mol/L) under refluxing. Sodium citrate tribasic dihydrate solution in DI water (4 × 10^−2^ mol/L) is added to the solution. Reaction completion is detected by color change from pale yellow to wine red. Boiling condition is further maintained for 15 min after color change is completed and then the solution is cooled to room temperature. The AuNP solution is dialyzed overnight using the Spectra/Por®7 membrane (1,000 Da cut) against DI Milli-Q water to remove excess sodium citrate tribasic dehydrate. Then, the solutions are placed on a typical copper grid followed by drying under air at room temperature for TEM (JEOL Cs-corrected HR-TEM, JEM-2200FS). Given that the organic layers of the AuNPs are not clearly detected by the electron beam in TEM, gold cores with high electron density in each AuNP are captured distinctively. From this TEM image, the average diameter of the formed AuNPs is determined to be approximately 20 nm.


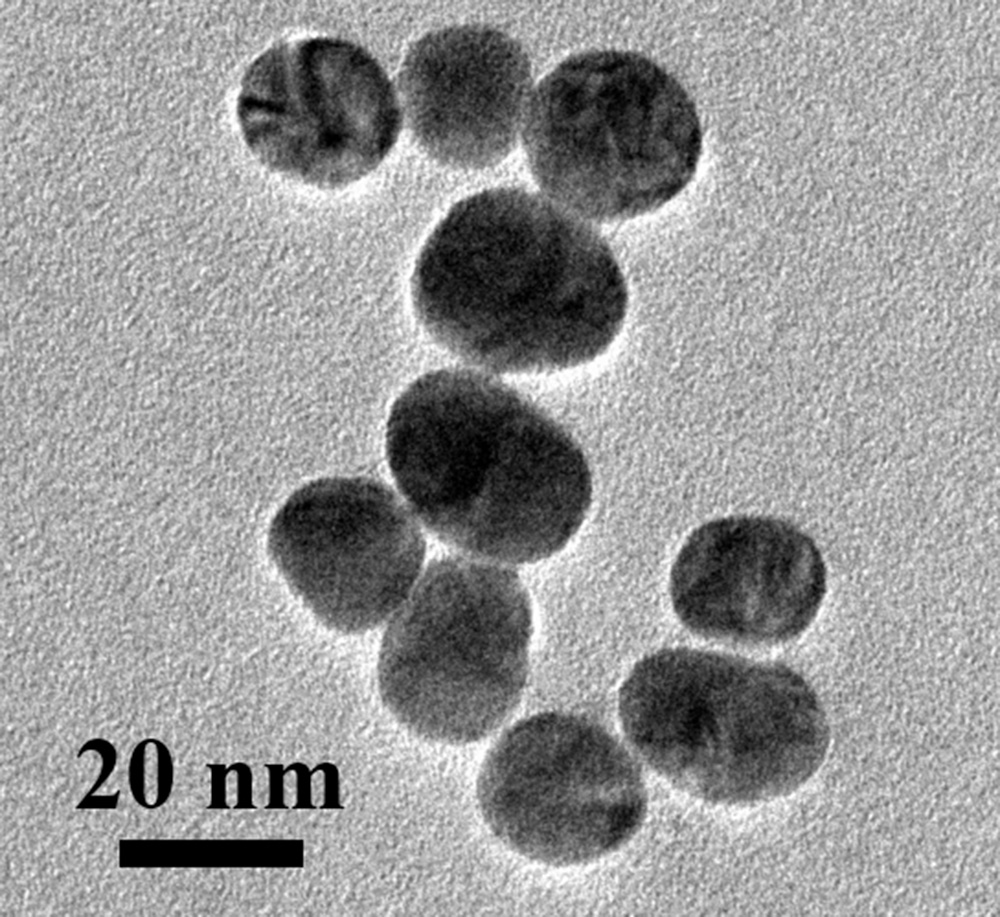


**Figure S1**. Typical TEM image of the citrate-covered AuNPs designed in this study.

**Preparation of ligand-coated AuNPs.** Thiol end-capped PEG ligands are added in the second step. After the size of the AuNP is determined using the aforementioned method, a designed amount of ligand aqueous solution is added to the aqueous solution to modify the surface properties of the citrate-covered AuNP and stirred at room temperature or higher (between 50 °C and 60 °C) for 6 h to 12 h until no further color change is observed. The unreacted residue ligands are minimized to less than 1 ppm by testing aliquots of the samples. The AuNP solution is dialyzed overnight using the Spectra/Por®7 membrane (25 KDa cut) against Milli-Q water for purification. The aqueous surface-modified AuNP stock solutions are adjusted to have a concentration of approximately 2.4 × 10^15^ AuNPs/L in consideration of the diameter of AuNPs (20 nm in average diameter) and the HAuCl_4_ concentration of 1.0 mmol/L. Given the standard concentration of the AuNP stock solution, the concentration of the ligand stock solution is controlled to be 1 mmol/L (~6 × 10^20^ ligands/L = ~2.5 × 10^5^ ligands/AuNP).


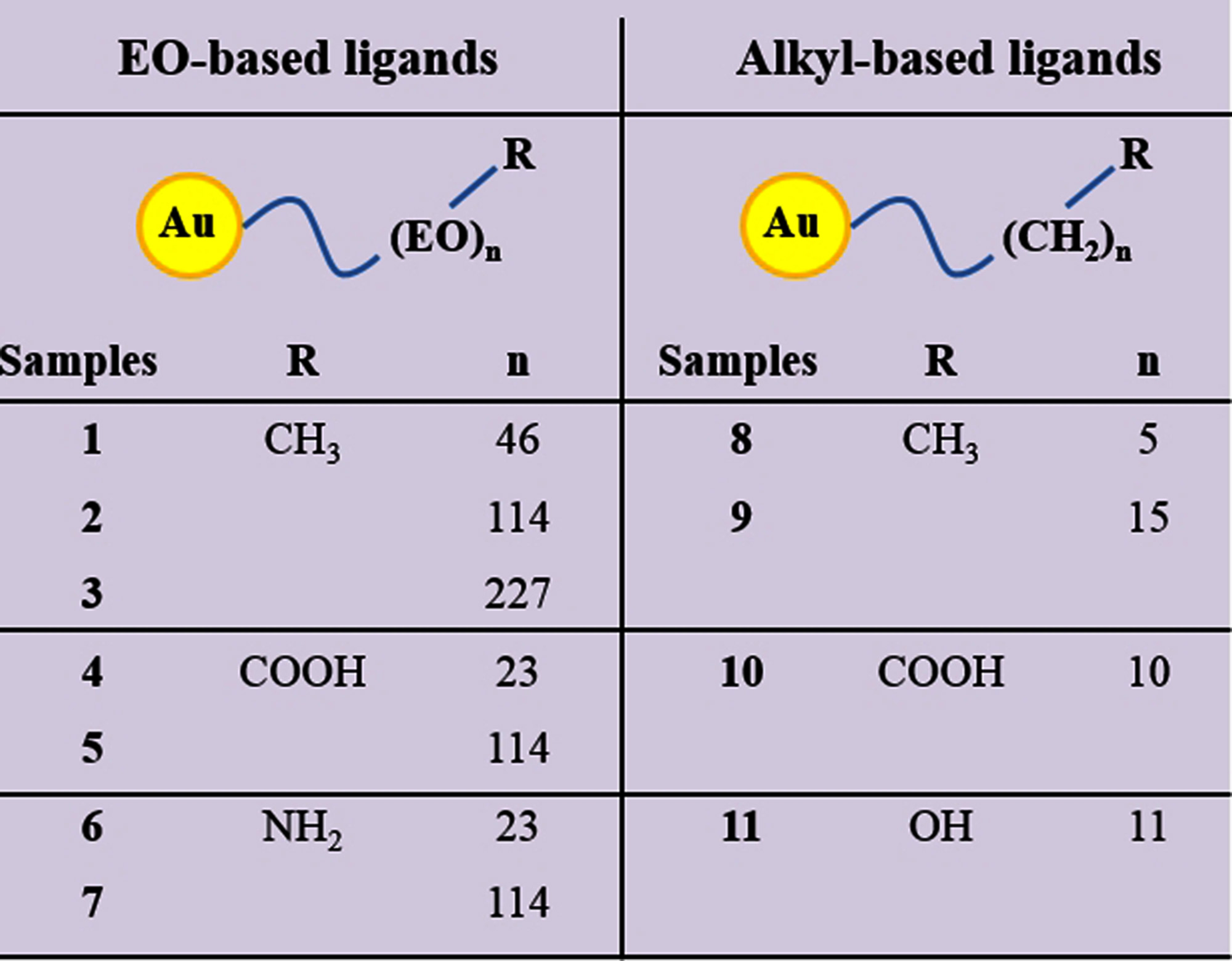


**Figure S2**. Ligand-covered AuNPs designed in this study.

**Figure S3.** SAXS results of the methyl end-capped PEO-conjugated AuNPs. The molecular weight of the PEO solution is controlled to be *n* = 46, *n* = 114, and *n* = 227 of the inset figures. **(a)** At a dilute condition where the structure factor approaches zero, the detected *q_I_* shifts to the higher *q* region as the EO unit number increases. **(b)** *q_I_* shifts to *q_II_* and *q_III_*, which are newly formed peaks by the aggregated structure formation for all three PEG systems.

**(a)**

**
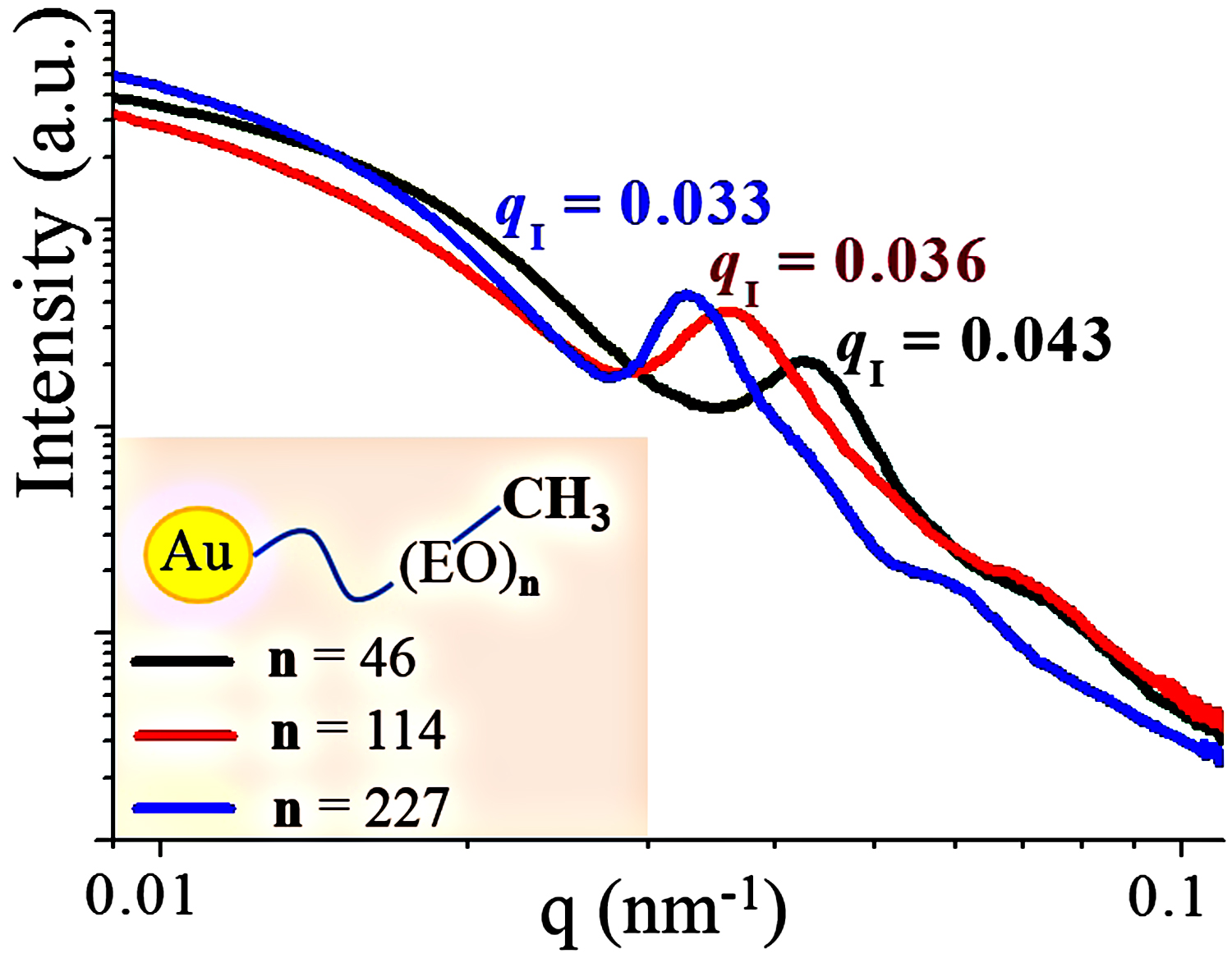
**

**(b)**

**
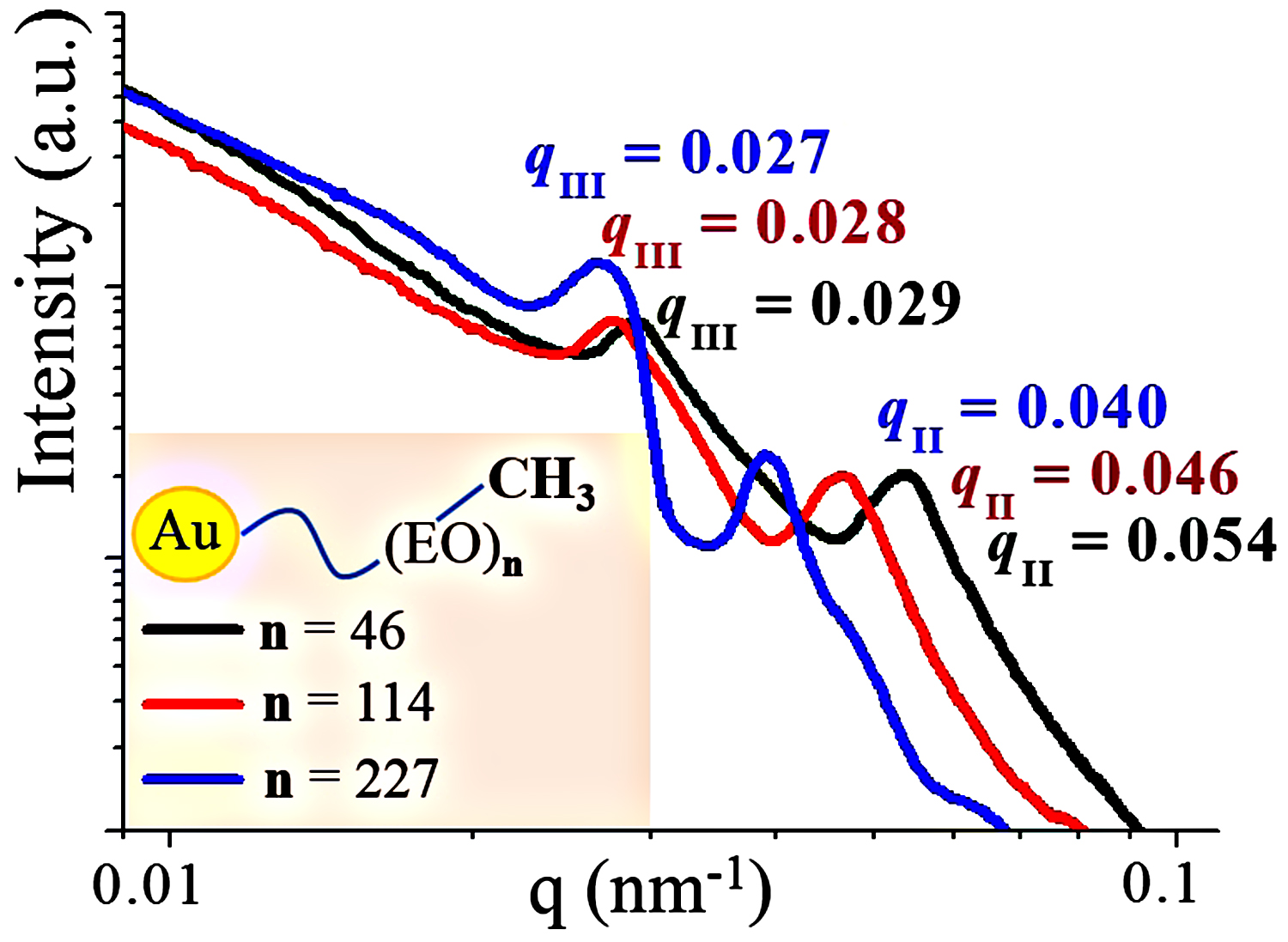
**

**Figure S4.** SAXS results of the PEG 10000-conjugated AuNP. The detected *q_I_* for the designed PEO 10000-anchored AuNP exhibits typical LCST, where the increase in temperature from 20 °C to 60 °C induces shrinkage of the PEO shell. The detected *q_I_* reflects the hydrodynamic size (*D_H_*) of the PEO 10000-anchored AuNP at a dilute condition (structure factor approaches zero), as depicted in Fig. 3c.

**
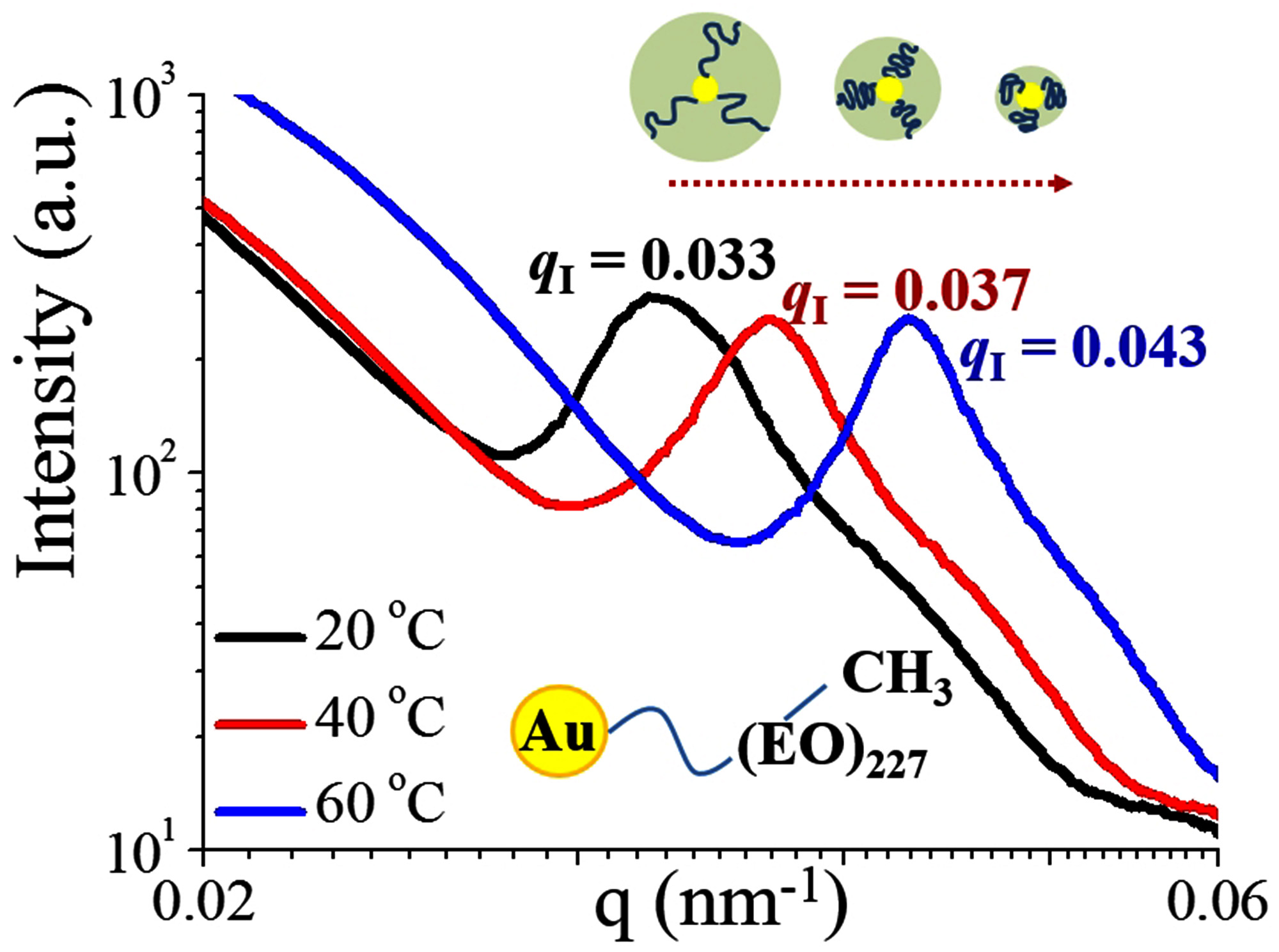
**

**Small-angle X-ray scattering (SAXS).** SAXS measurements are performed at the 4C synchrotron SAXS beam line of PAL. Samples of 1 mm thickness are used by stacking five Si wafer sample holders having 200 μm-thick SiN_3_ window at the center. Synchrotron SAXS measurements are performed at 4C beam line of PAL equipped with a position-sensitive two-dimensional (2D) detector. Samples of 1 mm thick are used by stacking the five 200 μm-thick Si wafer of SiN_3_ sample window. The sample-to-detector distance (SDD) as of 7 m covers the *q* range of 0.005 nm^−1^ < *q* < 0.15 nm^−1^, where *q =* (4π/λ)sin(θ/2) is the magnitude of the scattering vector and θ is the scattering angle. 4 m covers the *q* range of 0.0679 nm^−1^ < *q* < 1.64094 nm^−1^. The *q* range is calibrated using polystyrene-*block*-poly(ethylene-*ran*-butylene)-*block*-polystyrene (SEBS) (*q* = 0.19165 nm^−1^). On the other hand, the 1m SDD covers the *q* range of 0.346 nm^−1^ < *q* < 7.68039 nm^−1^. The *q* range is calibrated using silver behenate (*q* = 1.052 nm^−1^). All the measurements are performed at 25 °C isothermal condition. A W/B4C double multilayer monochromator are installed to deliver monochromatic X-rays with 6.75 nm (18360 keV) wavelength and spread of Δλ/λ = 0.01. The 2D scattered X-rays are recorded by a CCD camera (Mar CCD, Mar USA, Inc., CCD165). The collected SAXS data are corrected by subtracting the data of background and empty cell scattering. All measurements are performed at the isothermal condition of 20 °C.


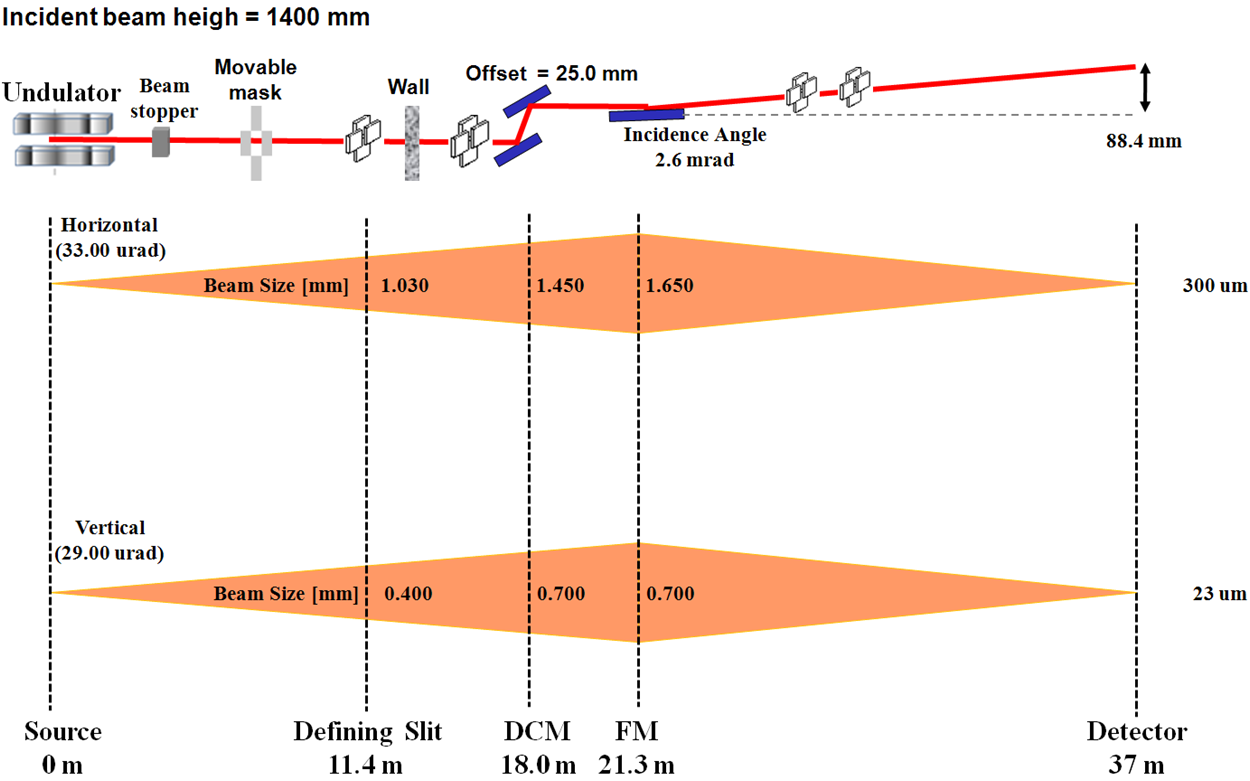


**Figure S5.** Experimental set-up for small angle X-ray scattering (SAXS) at PAL

**Synchrotron X-ray nanoscopy (XN)***.* Fig. S5 shows the optical layout of the 7C XNI beamline along with a photograph of the main components. High-ﬂux X-rays are provided by means of a 1.4 m-long hybrid-type in-vacuum undulator with a period of 20 mm. In order to focus, or rather collimate, the divergent X-rays, ten beryllium parabolic CRLs of diameter 1 mm and effective aperture 0.6 mm (reduced by absorption), positioned 25.7 m downstream from the source, are employed. In order to reduce the spatial coherency and homogenize the illumination, a diffuser (rotating paper) is inserted in front of the sample. The sample is mounted on a three-axis piezo-driven scanning stage on top of an air-bearing rotation stage. The objective zone plate, of 50 nm outermost zone width, 140 μm diameter and 1.0 μm thickness, is made of tungsten. For Zernike phase contrast, a holed aluminium-ﬁlm phase plate of 3.78 μm is positioned near the back focal plane of the zone plate. The thickness is selected so as to phase shift the diffracted beam by π/2 and, thereby, make the sample image darker in the bright ﬁeld. The hole, of 10 μm diameter, is drilled by focused ion beams. The detector comprises a thin (18 μm) Tb:LSO scintillator crystal of 10 mm diameter and an X20 homemade optical microscope. The microscope is composed of an X20 objective lens and a CCD. The CCD has 4096 × 4096 pixels of 9 μm size. All of the optics, including the diffuser, pinhole, sample, zone plate, phase plate and detector, are installed on a 5 m-long 30 cm-thick granite plate to minimize external vibration.


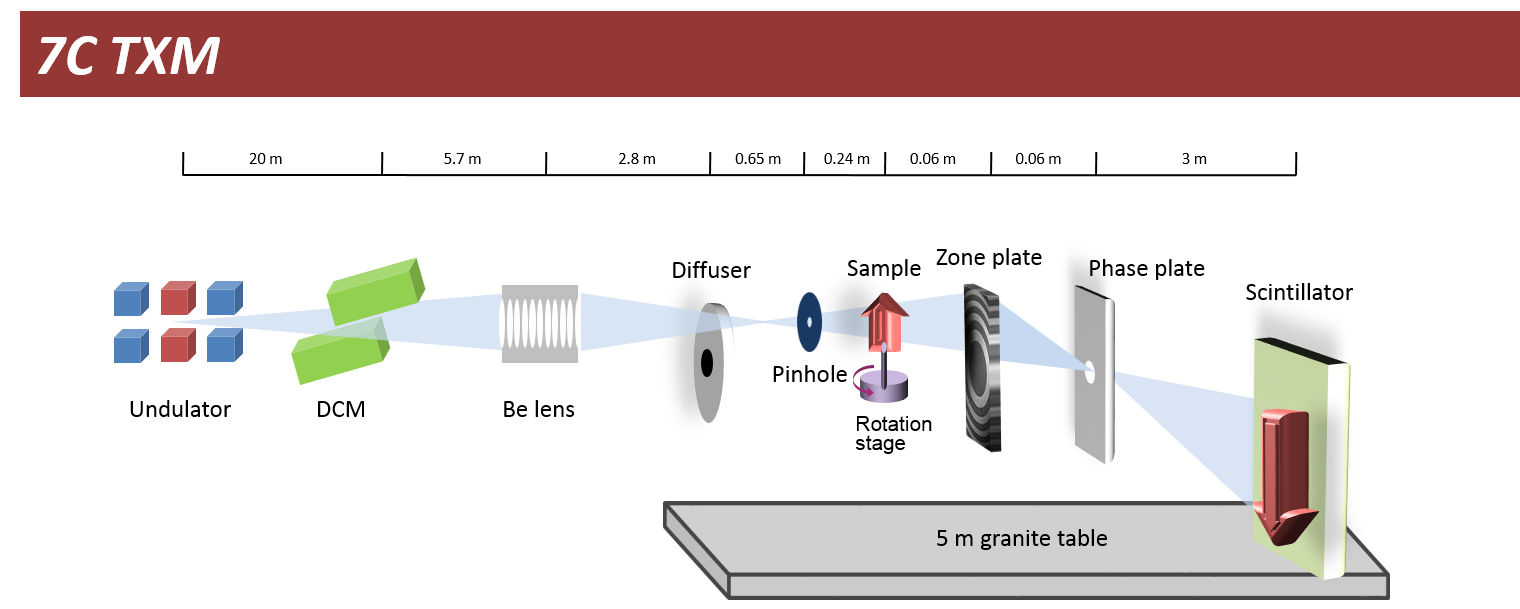


**Figure S6.** Experimental set-up for X-ray nanoscopy (XN) at PAL

**Two-dimensional (2D) X-ray microscopy (XM).** Synchrotron X-ray images are captured at 6D beam line of the Pohang Accelerator Laboratory (Pohang, Korea). The X-ray source was a bending magnet with a critical energy of 8.7 keV at 3GeV electron energy operation. The white beam was attenuated by a polished beryllium (Be) of 0.5 mm thickness or polished Si wafer of 1 mm thickness. The photon energy (E) was about 22 keV with energy resolution (ΔE/E) of 15.8 keV to 34.4 keV (~ 84 %). The brightness was 22 × 10^12^ (ph/s/mm^2^) and the beam size was about 30 mm (H) × 5 mm (V). The samples were placed at approximately 30 m downstream of the source, whereas the detector was placed downstream of the objects at 30 cm. The size of the X-ray beam illuminating the test sample was adjusted to that of the field-of-view using a slit module to avoid unnecessary exposure of X-ray beam on the sample. An attenuator made of aluminum (Al) sheets was located at the beam inlet of the experiment hutch on the way of X-ray propagation pathway to attenuate light intensity, even when no image was photographed. This attenuation of light intensity protected the sample and detector from strong X-ray irradiation. The primary X-ray image was converted into a visible image on a thin scintillator crystal CdWO4 of 100 μm thickness. X-ray images were captured using a CCD camera (PCO, PCO2000). The field-of-view with a 10× objective lens attached in front of the camera was approximately 3.6 mm × 2.4 mm in physical dimension. The pixel size was about 0.9 μm.


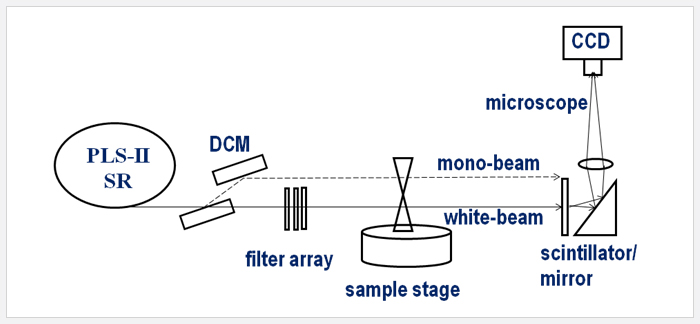


**Figure S7.** Experimental set-up for X-ray microscopy (XM) at PAL.
